# Uranium-rich diagenetic fluids provide the key to unconformity-related uranium mineralization in the Athabasca Basin

**DOI:** 10.1038/s41598-019-42032-0

**Published:** 2019-04-02

**Authors:** Guoxiang Chi, Haixia Chu, Duane Petts, Eric Potter, Simon Jackson, Anthony Williams-Jones

**Affiliations:** 1 Department of Geology, University of Regina, Regina, Saskatchewan Canada; 2 China University of Geosciences, Beijing, China; 3 Geological Survey of Canada, Ottawa, Ontario Canada; 4 Department of Earth and Planetary Sciences, McGill University, Montreal, Canada

## Abstract

The Proterozoic Athabasca Basin is well known for its unusually large-tonnage and high-grade ‘unconformity-related’ uranium (U) deposits, however, explanations for the basin-wide U endowment have not been clearly identified. Previous studies indicate that U-rich brines with up to ~600 ppm U and variable Na/Ca ratios (from Na-dominated to Ca-dominated) were present at the sites of U mineralization, but it is unknown whether such fluids were developed solely in the vicinity of the U deposits or at a basinal scale. Our microthermometric and LA-ICP-MS analyses of fluid inclusions in quartz overgrowths from the barren part of the basin indicate that U-rich brines (0.6 to 26.8 ppm U), including Na-dominated and Ca-dominated varieties, were widely developed in the basin. These U concentrations, although not as high as the highest found in the U deposits, are more than two orders of magnitude higher than most naturally occurring geologic fluids. The basin-scale development of U-rich diagenetic fluids is interpreted to be related to several geologic factors, including availability of basinal brines and U-rich lithologies, and a hydrogeologic framework that facilitated fluid circulation and U leaching. The combination of these favorable conditions is responsible for the U fertility of the Athabasca Basin.

## Introduction

Uranium (U) is a trace element with an average crustal abundance of 1.7 ppm (0.5 ppm for oceanic crust and 2.7 ppm for upper continental crust)^1^. The minimum grade for economic exploitation of U is ~0.03 wt.% U^2^ (for sandstone-hosted deposits), which is about 170 times the average crustal value. However, most ‘unconformity-related’ U deposits associated with Proterozoic sedimentary basins have average grades of >0.3 wt.% U, with many >2 wt.% U^2,3^. Several giant U deposits in the Athabasca Basin in northern Canada have average grades above 10 wt.% U, including the McArthur River (14.87 wt.% U_3_O_8_ - 345.2 million pounds of U_3_O_8_^4^), Cigar Lake (17.84 wt.% U_3_O_8_ - 234.9 million pounds of U_3_O_8_^4^), Arrow high-grade core (18.84 wt.% U_3_O_8_ - 164.9 million pounds of U_3_O_8_^5^) and Phoenix (19.1_3_ wt.% U_3_O_8_ - 71.3 million pounds of U_3_O_8_^6^) deposits, which are enriched by up to 100,000 times the crustal value. These deposits and many others like them in other Proterozoic basins have been the subject of intense study by large numbers of researchers, and different models have been proposed^7–9^, but it is still not well understood why these basins, particularly the Athabasca Basin, are so richly endowed in U.

The ore-forming fluids of the unconformity-related U deposits have been shown to be brines with high concentrations of U (up to 600 ppm U) based on analysis of fluid inclusions from the U deposits in the Athabasca Basin^10–12^. Halogen and noble gas geochemistry^13–15^ and B isotope signatures in tourmaline associated with the U mineralization^16^ suggest that the ore-forming brines are of seawater evaporation origin, although an alternative origin from dissolution of evaporites also has been proposed^17^. The source of the U has been controversial, with opinion divided over whether the U was mainly derived from detrital minerals in the basin^8,18–22^, or mainly from the underlying basement rocks^9,10,23–27^. The argument for a basin source of U is based mainly on the oxidizing nature of the sediments (as indicated by the development of red beds), which is favorable for U dissolution and transport. The argument for a basement source for U, on the other hand, is built on several observations including: (1) U concentrations are generally higher in the basement rocks than in the currently preserved sedimentary rocks (mainly sandstones) in the basin; (2) there are more leachable U in the basement (e.g., uraninite and monazite)^24,25^; (3) some U deposits are located up to ~1 km below the unconformity (e.g., Arrows^5^); (4) percolation of basinal brines into the basement were recorded by fluid inclusions^28^, and (5) many of the fluid inclusions from the U deposits contain Ca-dominated brines, which could not be derived from the basin because the sandstones contain low concentrations of Ca^10,26,27^.

In order to establish whether the U was extracted from the basin and/or basement, it is necessary to have direct evidence indicating that U-rich brines were actually present within the basin and/or basement. Such evidence can be captured in fluid inclusions that recorded passage of diagenetic fluids in the basin and/or fluids in the basement (which ultimately may have been derived from the basin), in areas far away from known U deposits. Here, we report the first direct evidence of the extensive development of U-rich diagenetic fluids in the Athabasca Basin. This evidence was derived from Laser Ablation - Inductively Coupled Plasma - Mass Spectrometry (LA-ICP-MS) analysis of fluid inclusions entrapped in quartz overgrowths on detrital quartz grains. Based on these data, we propose that intense reaction between basinal brines and sediments prior to significant compaction and cementation, and perhaps including the uppermost part of the basement, was essential for the basin-scale U mineralization.

**Figure 1:**
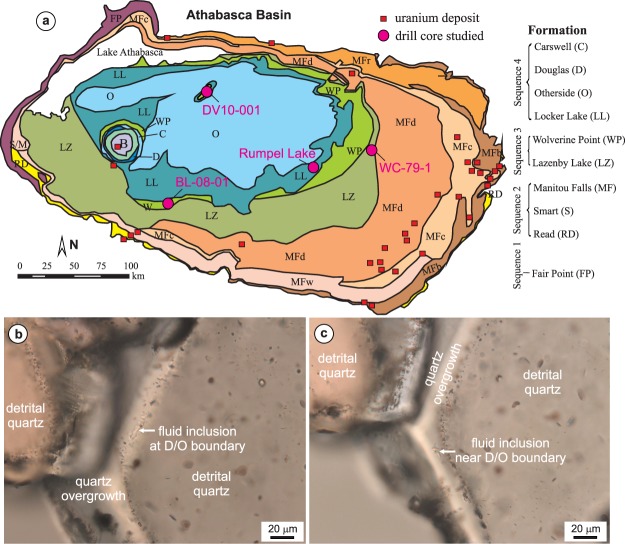
(**a**) Geological map of the Athabasca Basin in northern Saskatchewan (Canada) showing the location of the drill holes from which the core samples used in this study were collected; (**b**) fluid inclusions along the detrital-overgrowth boundary; (**c**) a fluid inclusion near the detrital-overgrowth boundary.

The Athabasca Basin is filled with Paleo- to Mesoproterozoic sedimentary rocks that, from bottom to top, are divided into the Fair Point, Smart/Read, Manitou Falls, Lazenby Lake, Wolverine Point, Locker Lake, Otherside, Douglas, and Carswell formations^29^. These units consist of fluvial sandstone, with the exception of the Wolverine Point Formation (marginal marine mudstone/siltstone/sandstone), Douglas Formation (marine mudstone/siltstone/shale), and Carswell Formation (marine carbonates). Most of the sedimentary rocks (except the Douglas and Carswell formations) comprise red beds with variable degrees of bleaching and cementation^30^. The U deposits are associated with reactivated basement faults crosscutting the unconformity between the basin and the basement^3^. The samples examined in this study are sandstones from the Read, Manitou Falls, Lazenby Lake and Wolverine Point formations, and were collected from four drill cores that are distal from known U mineralization (Fig. 1a).

## Results

**Figure 2:**
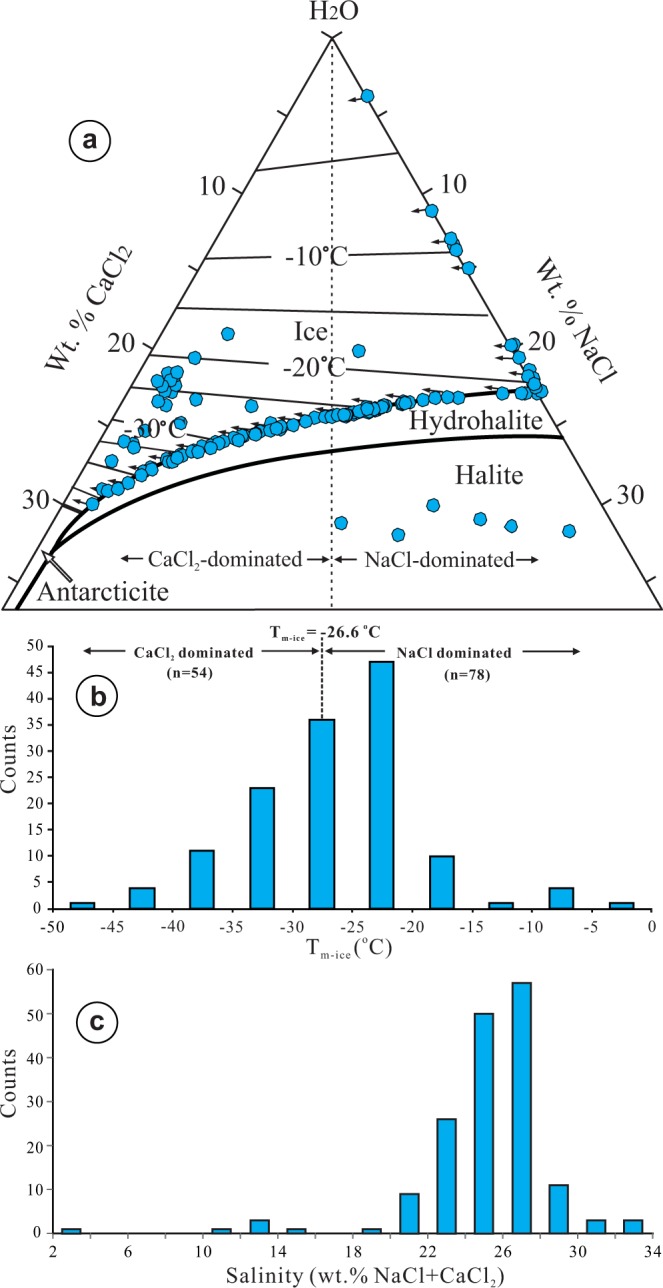
(**a**) A H_2_O-NaCl-CaCl_2_ phase diagram showing the composition of the fluid inclusions from quartz overgrowths (base diagram constructed using the program of Steele-MacInnis *et al*.^50^). The arrows indicate that the true composition of the inclusions is shifted in that direction; (**b**) A histogram of ice-melting temperatures of the fluid inclusions; (**c**) A histogram of salinities of the fluid inclusions.

Fluid inclusions were examined in quartz overgrowths on detrital quartz grains in the sandstones. Most of them occur along (Fig. 1b) or near (Fig. 1c) the detrital-overgrowth boundaries and are irregularly shaped, with the longest dimension ranging from a few microns up to 24 microns. The majority of the inclusions are composed of a liquid and a vapor at room temperature, with the vapor mostly comprising ~10 vol. % of the inclusions. The homogenization temperatures range from 50° to 212 °C (Supplementary Table 1). A few (6 out of 158) of the fluid inclusions contain a halite crystal in addition to liquid and vapor (Fig. 2a; Supplementary Table 1); the halite crystal is interpreted to be an accidently entrapped solid rather than a daughter mineral, based on the coexistence of halite-bearing and halite-free inclusions. Many of the fluid inclusions did not freeze during cooling and most of those that were frozen begin to melt at temperatures <−50 °C. These characteristics, together with Raman spectroscopic data for frozen inclusions indicating the presence of NaCl and CaCl_2_ hydrates, suggest that the fluid composition can be represented by the H_2_O-NaCl-CaCl_2_ system. Of the 132 fluid inclusions that were frozen, 110 inclusions have ice-melting temperatures <−21.2 °C (eutectic point of the H_2_O-NaCl system) (Supplementary Table 1), further supporting the interpretation of a H_2_O-NaCl-CaCl_2_ fluid.

The composition of fluid inclusions that yielded both ice-melting and hydrohalite-melting temperatures (26 out of 132) is mainly in the CaCl_2_-dominated field (to the left of the dashed line in the ice field in Fig. 2a). For fluid inclusions only yielding ice-melting temperatures >−21.2 °C, the composition may fall anywhere on the ice-melting isotherm left of the intersection between the isotherm and H_2_O-NaCl binary, as indicated by the arrows pointed away from the data points on the H_2_O-NaCl binary (Fig. 2a). For fluid inclusions only yielding ice-melting temperatures <−21.2 °C, the compositions may fall anywhere on the ice-melting isotherm left of the intersection between the isotherm and the ice-hydrohalite cotectic curve, as indicated by the arrows pointed away from the data points on the ice-hydrohalite cotectic curve (Fig. 2a). It is evident that about half of the fluid inclusions are CaCl_2_-dominated and half are NaCl-dominated (Fig. 2a). This is also reflected by the ice-melting temperatures (Fig. 2b): ice-melting temperatures <−26.6 °C (n = 54) indicate CaCl_2_-dominated fluids, whereas ice-melting temperatures >−26.6 °C (n = 78) may indicate either CaCl_2_-dominated or NaCl-dominated fluids depending on the hydrohalite-melting temperature. Most of the fluid inclusions have salinities between 20 and 30 wt.% NaCl + CaCl_2_ (Fig. 2c; Supplementary Table 1).

**Figure 3:**
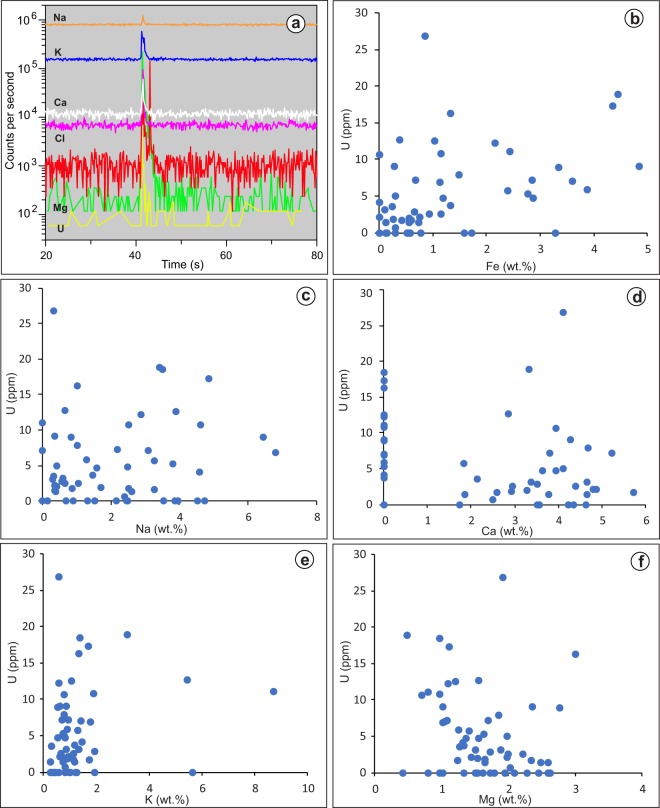
(**a**) LA-ICP-MS signals for the various elements detected in a fluid inclusion from quartz overgrowth; (**b**–**f**) Plots showing the concentration of U versus Fe, Na, Ca, K, and Mg in the fluid inclusions.

Sixty-eight (68) of the fluid inclusions analyzed microthermometrically were selected for LA-ICP-MS analysis. Fifty-five (55) of these inclusions displayed prominent Cl peaks as well as peaks for Na, Ca, Mg, K and Fe (Fig. 3a; Supplementary Table 2). Some of the ablations recorded a secondary Fe peak, without corresponding Cl and Na or Ca peaks (Fig. 3a), which is considered to have originated from nearby iron oxide inclusions. A second, much smaller U peak was also associated with the secondary Fe peak (Fig. 3a), which may indicate that a minor amount of U was associated with the iron oxide inclusions. However, the secondary Fe peaks (and minor U) were excluded during data processing.

**Figure 4:**
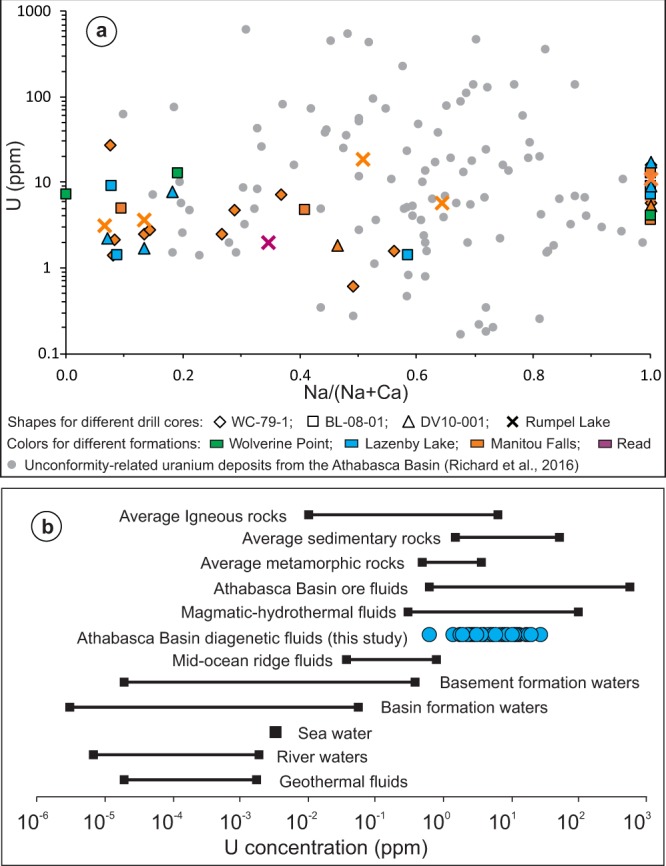
(**a**) The concentration of U as a function of the Na/(Na + Ca) ratio for fluid inclusions in quartz overgrowths considered in this study and fluid inclusions in U deposits in the Athabasca Basin (data from Richard *et al*.^12^); (**b**) Ranges of U concentrations for various rocks and naturally occurring geofluids (data sources shown in Supplementary Table 3).

Forty (40) of the inclusions have elevated U concentrations (associated with Na, Cl, Ca, Mg and K peaks), which range from 0.6 to 26.8 ppm and average 6.8 ppm U (Supplementary Table 2). The ranges and average concentrations of the major solutes are: 1,656–67,899 (avg. 21,875) ppm Na, 17,288–57,004 (avg. 36,396) ppm Ca, 4,214–29,951 (avg. 16,178) ppm Mg, 2,601–87,031 (avg. 12,685) ppm K, and 907–48,282 (avg. 14,124) ppm Fe (Supplementary Table 2). No correlation was observed between U and the major elements (Fig. 3b–f). Of the 54 fluid inclusions analyzed for Na and Ca, 27 have Na/(Na + Ca) ratios <0.5 (Ca-dominated), and 27 inclusions have Na/(Na + Ca) ratios >0.5 (Na-dominated) (Supplementary Table 2). There is no correlation between U and Na/(Na + Ca) ratios, nor between them and sample locations or stratigraphic positions (Fig. 4a).

The U concentrations of fluid inclusions obtained in this study (0.6 to 26.8 ppm) fall in the lower part of the ranges of ore-forming fluids of unconformity-related U deposits and magmatic-hydrothermal fluids (Supplementary Table 3)^11^, and are more than two orders of magnitude higher than those of most naturally occurring geologic fluids (Fig. 4b). Both the U and the Na/(Na + Ca) ratios overlap with the U and the Na/(Na + Ca) ratios of the majority of fluid inclusions from the U deposits (Fig. 4a).

**Figure 5:**
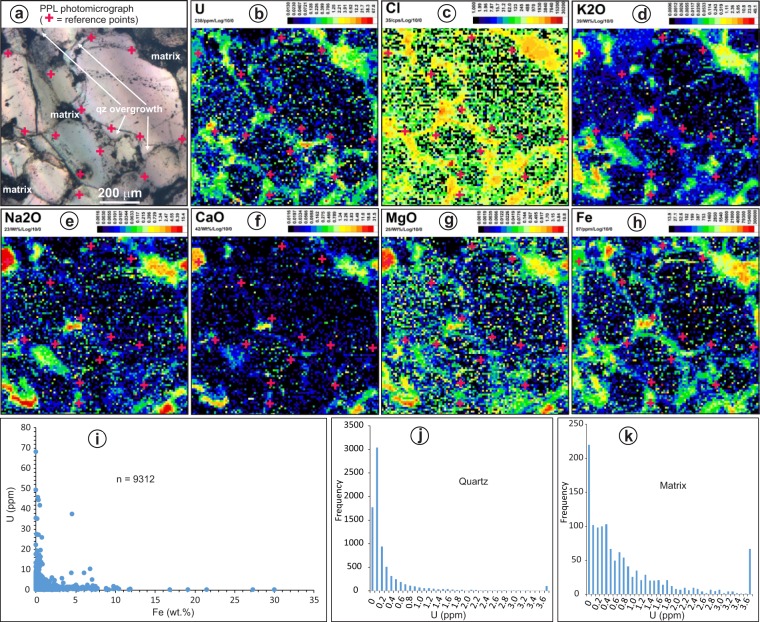
(**a**) Photomicrograph of detrital quartz, quartz overgrowths and matrix in an Athabasca sandstone; (**b**–**h**) LA-ICP-MS maps of the area in a) showing the distribution of U, Cl, K, Na, Ca, Mg and Fe. The color patterns (log scale) illustrate quantitative values (wt.% or ppm) calculated based on 100% normalization of all elements. It was not possible to quantify Cl during the mapping session, and values are reported as raw cps (counts per second); (**i**) plot of U and Fe of all LA-ICP-MS analytical points for the mapping area shown above; (**j**) histogram showing the distribution of U concentrations in quartz; (**k**) histogram showing the distribution of U concentrations in the matrix.

LA-ICP-MS mapping of part of a sandstone thin section (Fig. 5a) indicates that the major elements in the fluid inclusions (Na, Ca, K, Mg, Fe and Cl) are relatively enriched in the matrix between detrital quartz grains (Fig. 5c–h), which is mainly made of kaolinite, illite and Fe oxides. Uranium is also enriched in the matrix (Fig. 5b). However, concentrations of these elements are low in the quartz overgrowths (compare Fig. 5a to Fig. 5b–h using the reference points) as well as in the detrital quartz. Data from the LA-ICP-MS mapping (9312 points) indicate that all the points with >10 wt.% Fe are low (<1 ppm) in U, and all the points (except two) with >10 ppm U have <1 wt.% Fe (Fig. 5i), indicating that Fe oxides are not the main U-carrying minerals. The dataset also indicates that quartz mostly contains <0.2 ppm U (Fig. 5j), and most of the matrix contains <1 ppm U (Fig. 5k).

## Discussion

Both the microthermometric and LA-ICP-MS data indicate that the Na-dominated and Ca-dominated brines, which have been documented in unconformity-related U deposits^10–12,26,27^, were widely distributed in the Athabasca Basin at a variety of stratigraphic levels and in locations far from mineralized areas. The LA-ICP-MS data indicate that these basinal brines were uraniferous, and compositionally similar to fluid inclusions from the U deposits in terms of U contents and Na/(Na + Ca) ratios (Fig. 4a), although the highest U values reported for the deposits have not been found in the diagenetic fluids. Although analysis of some of the inclusions could have been compromised by contamination with Fe oxides (for Fe) and clay minerals (for K) present along the boundary between detrital quartz and quartz overgrowths, the lack of correlation between U and Fe or K concentrations (Fig. 3b–f) indicates that the U signal was mostly contributed by the fluid inclusions. The L-shaped correlation between U and Fe in the LA-ICP-MS mapping dataset (Fig. 5i), i.e., highest Fe corresponding to low U and highest U corresponding to low Fe, further supports that Fe oxides are not the main U-carrying minerals.

The relative enrichment of U, Cl, Na, Ca, K, Mg and Fe in the matrix as revealed by LA-ICP-MS mapping (Fig. 5) also testifies to the presence of the uraniferous basinal brines in the sandstones, although the timing of the mobilization could not be determined by this type of analysis. However, the occurrence of uraniferous brine inclusions along or near the detrital-overgrowth boundary indicates that the uraniferous brines were present throughout the basin when the sandstones had not fully compacted and/or been cemented. A comparison of trace element concentrations between red sandstones and bleached counterparts suggests that during bleaching, which took place when the sandstones were fully compacted, the U content in the diagenetic fluid was low^30^.

The high concentrations of Mg and K in the fluid inclusions (Supplementary Table 2) relative to seawater (avg. 16,178 vs. 1,294 ppm Mg, and avg. 12,685 vs. 399 ppm K), which were also observed in fluid inclusions from the U deposits^10–12^, support a seawater evaporation origin for the brines^31,32^. A similar origin for basinal brines was proposed for the Kombolgie Sub-basin in Australia, which also hosts unconformity-related U deposits^22,33,34^. Although the bulk of the Athabasca Basin sediments were deposited in a continental environment^29^, the presence of gypsum pseudomorphs and solution-collapse breccias in the stromatolitic dolomite of the Carswell Formation suggests the development of marine evaporites^35^. Accordingly, it is inferred that the brines recorded throughout the Athabasca Basin were produced during the deposition of the Carswell Formation and succeeding strata (now eroded), as also has been proposed by Richard *et al*.^13–15^. Based on regional geochronology and stratigraphic data, it was proposed that the primary U mineralization in the Athabasca Basin took place at relatively shallow depths (<~3 km) during the deposition of the Carswell Formation^36^, at which time the sediments in the basin had not been significantly compacted and cemented, in contrast to a conventional model suggesting deep burial (>5 km) environment for mineralization^26,37^.

Because brines derived from seawater evaporation are characterized by low Ca concentrations compared to Na, Mg and K^31^, Ca-rich brines in sedimentary basins most likely resulted from compositional exchange with Ca-bearing minerals^32^ during diagenesis, although the initial Ca content in the evaporated seawater brine may vary due to secular changes of seawater chemistry^38^. Possible mechanisms to explain the Ca-rich brines in the Athabasca Basin include dolomitization of carbonates of the Carswell Formation, alteration of Ca-bearing minerals such as feldspars, apatite and titanite in the sandstones, and alteration of Ca-bearing minerals in the uppermost part of the basement. The first mechanism may be important as the Carswell Formation, which is up to 500 m thick, is made of dolomitized carbonates^29^. In comparison, the contribution of the second mechanism may be relatively small, because the sandstones that are currently preserved in the Athabasca Basin are poor in Ca-bearing minerals, with quartz accounting for more than 99% of the framework grains^3^. Any detrital feldspars that were initially in the sandstones may have been altered to clay^39,40^. Considering that clay minerals form less than 3 vol % of the sandstones in the Athabasca Basin^22^, the maximum amount of feldspars in the sandstones before diagenesis was likely less than 3 vol %. The third mechanism requires a fluid convection system in which basinal brines infiltrated into the upper part of the basement and circulated back into the basin^27^. Such a fluid convection system is theoretically possible based on numerical modeling^41,42^, and may have been further facilitated by elevated geothermal gradients as implied in the shallow-burial model^36^ discussed above. However, the efficiency of this mechanism depends on the permeability of the upper part of the basement, which remains poorly constrained.

Similar to Ca, the high concentrations of U found in the diagenetic fluids may have been derived from multiple sources, including strata above the preserved sandstones, within the preserved sandstones, and the upper part of the basement. However, the lack of correlation between U and Ca concentrations in the brine inclusions (Figs 3d and 4a) suggests that they may not have been derived from the same source rocks. The U contents of sedimentary rocks are highly variable, ranging from 0.01–0.43 ppm for evaporites, 2.2 ppm for average carbonate rocks, 0.45–3.2 ppm for sandstones, 3.7 ppm for common shales, 3–1250 ppm for black shales, and 50–300 ppm in phosphate rocks^1^. Based on the data of Wright *et al*.^43^ and Chu *et al*.^30^, most of the sedimentary rocks currently preserved in the Athabasca Basin contain <1.5 ppm U. It is likely that the initial U contents of the sediments in the basin, perhaps present in U-bearing accessary minerals such as zircon, monazite, xenotime, apatite and titanite, were higher than the current levels, and U was leached during diagenesis. Like Ca, some U may have also been leached from the upper part of the basement and circulated back into the basin through fluid convection.

Despite the uncertainties about the specific sources of U and Ca as discussed above, the finding of U-rich diagenetic fluids within the basin provides key evidence towards deciphering why the Athabasca Basin is favorable for U mineralization. Although basinal brines are key to the formation of the unconformity-related U deposits^10–16^, the availability of basinal brines alone is insufficient for U mineralization. Instead, the combination of the availability of U-rich source rocks and basinal brines, as well as a hydrogeologic setting that allows the basinal brines to react extensively with the source rocks, may be the determining factor for basin-scale U mineralization. Such conditions appear to be satisfied in the Athabasca Basin, as discussed below.

Firstly, U-rich lithologies are widely distributed in the basement of the Athabasca Basin^7,23–25^, and the sediments within the basin, which were derived from similar rocks in the provenance areas, may have inherited some of the U in the source rocks^19–21^. These provide the material basis for later U mineralization. Secondly, the paleo-geographic position of the Athabasca Basin is suitable for the development of basinal brine. At the time of deposition of the Carswell Formation, the Athabasca Basin was located within 30° of the equator^44,45^, which provided ideal paleo-environmental conditions for the development of brines through seawater evaporation^13^. The high salinity and possible low pH of the brines^11^, combined with the oxidizing conditions in the basin, provided ideal conditions for the brines to extract U from the source rocks. Thirdly, the hydrogeologic framework of the basin was favorable for extensive reaction between the brine and the source rocks. The sandstone-dominated nature of the cover immediately above the basement and the hydrostatic fluid pressure regime associated with it^46,47^ were favorable for brine infiltration and fluid convection. Owing to their high density, the brines derived from seawater evaporation during deposition of the Carswell Formation would have refluxed into the underlying sediments^48^ and flushed out the low-salinity fluids initially occupying the interstitial pores of the fluvial sandstones. The U initially contained in the sediments was probably not leached significantly until the massive production of brine due to seawater evaporation during the Carswell period. Despite the large interval of time between the start of sedimentation in the basin (ca. 1720 Ma) and primary U mineralization (ca. 1540 Ma), the sediments probably remained poorly consolidated due to shallow burial^36^, which further facilitated brine migration and fluid-rock reaction. Finally, the high geothermal gradients implicated by the high fluid temperatures of ~200 °C^12,19,26,37^ and shallow burial (~3 km) environments as constrained by regional geochronological and stratigraphic data^36^, may have enhanced fluid convection^41,42^, which further facilitated fluid-rock reactions and extraction of U from source rocks including the uppermost part of the basement.

Thus, it is the coupling of several geologic conditions favoring U extraction that distinguishes the Athabasca Basin from most other sedimentary basins. The widespread development of U-rich diagenetic fluids in a basin, as revealed in this study for the Athabasca Basin, may be used as an indicator of the favorable U-leaching conditions. The studies of fluid inclusions in authigenic quartz in the sedimentary rocks, combined with analysis of potential chemical traps as well as basin and basement structures that may have channelled U-rich diagenetic fluids, provide a powerful tool for assessing the U fertility of a basin.

## Methods

Microthermometric measurements of fluid inclusions in quartz overgrowths were carried out using a Linkam THMS 600 Heating/Freezing stage attached to an Olympus BX51 petrographic microscope at the Geofluids Laboratory of the University of Regina. The stage was calibrated with synthetic standard fluid inclusions of H_2_O with an ice-melting temperature of 0 °C and critical temperature of 374.1 °C, and fluid inclusions of H_2_O-CO_2_ with a CO_2_-melting temperature of −56.6 °C. The precision of the ice melting temperatures was better than +/−0.2 °C. The program of Steele-MacInnis *et al*.^43^ was used to calculate salinity and NaCl/(NaCl + CaCl_2_) ratios.

The LA-ICP-MS analyses were conducted at the Geological Survey of Canada (Ottawa, Canada) using a Photon Machines Analyte G2 193 nm laser ablation system, equipped with a Helex dual-volume cell and a squid device, and coupled to an Agilent 7700x ICP-MS. An in-house solution was used as the primary calibration standard for the fluid inclusion analyses and was prepared using ICP-MS-grade, standard solutions of Na, Mg, Cl, K, Ca, Fe, Br and U. The solution standard was loaded into nylon capsules that were tightly covered in parafilm and placed within bored out spaces of a 1” Teflon mount. During the analytical session, measurements on NIST-610 glass were used to assess the solution standard and to evaluate instrument drift.

Analytical conditions for the inclusion analyses included a fluence of 5.3 J/cm^2^ (70% of 5 mJ), a spot size of 20 µm and a repetition rate of 10 Hz. For deep inclusions (greater than ~20 µm below the polished surface), a fluence of ~6.8 J/cm^2^ (90% of 5 mJ) was required to ablate deep enough into the host quartz. Each analysis consisted of a 40 s background measurement, 60 s of ablation and 50 s of washout between analyses.

SILLS data reduction software was used to calibrate the fluid inclusion data and convert the raw data (in cps) to concentrations, and to correct for instrument drift. A salt correction, based on charge balance, was applied to the fluid inclusion data using the calculated total salinity values determined using microthermometry and the signals for Na, Ca, Mg and K. Sodium was used as an internal standard for the calibration on NIST-610, based on the GeoReM preferred value (Na_2_O = 13.4 wt.%, updated in 2011).

The detection limit for U in the fluid inclusions was generally better than 1 ppm, whereas the propagated uncertainty for the U data was commonly within ±11 − 20%. The calculated values for NIST-610 were generally within 10% of the accepted values for Mg, K, Ca, Fe and U. An attempt was made to determine Cl and Br concentrations during the LA-ICP-MS sessions, however, most of the calculated concentrations fell below the detection limits of each analysis.

LA-ICP-MS mapping was conducted using a modified instrument setup that included the use of a 2 mm O.D. He and Ar carrier gas line (~1.5 m long), with a no signal mixing device that entered directly into the torch assembly. Washout times of approximately 300 ms (99.9% drop in signal intensity) were achieved using this carrier gas setup. The elemental maps were prepared using a series of linescans across the sample surface, using a fluence of 8.0 J/cm^2^ (88.1% of 6 mJ), a spot size of 10 µm, a repetition rate of 30 Hz and a scan speed of 5 µm/s. A pre-ablation pass was completed before every analysis to clean the sample surface. The raw, time-resolved signals were calibrated to linescan analyses of GSE-1G and were converted to concentrations using the 100% normalization method^49^. Data processing and compilation of concentration maps was undertaken using LAMtrace and pixeLAte data reduction software (versions 410 and 152, respectively). During the mapping session, absolute concentrations for Cl and Br could not be quantified due to their low abundance in GSE-1G, as such, values for these elements are reported in raw counts per second (cps).

In order to use the LA-ICP-MS mapping data to determine the concentrations of various elements in quartz and matrix separately, a threshold value of >80% SiO_2_ was selected to distinguish quartz from matrix after many trial-and-error tests. This threshold value produces the best fit of quartz grain – matrix distribution as constrained by petrography (Fig. 5a). The data extracted from the mapping dataset were then used to evaluate the potential effect of involvement of solid minerals (especially Fe oxides) on element concentrations (especially U) in fluid inclusions.

## Supplementary information

Supplementary information

## Data Availability

The datasets generated during and/or analyzed during the current study are available from the corresponding author on reasonable request.
