# Supplementary material for: Uranium-rich diagenetic fluids provide the key to unconformity-related uranium mineralization in the Athabasca Basin
Source: Sci Rep. 2019 Apr 2;9:5530. doi: 10.1038/s41598-019-42032-0 (PMC6445087; doi:10.1038/s41598-019-42032-0)
Supplement: Supplementary file 1 — Supplementary information [file 41598_2019_42032_MOESM1_ESM.pdf]

Uranium-rich diagenetic fluids provide the key to unconformity-related uranium mineralization in the Athabasca Basin

Guoxiang Chi[1 ID], Haixia Chu[1,2 ID], Eric Potter[3], Duane Petts[3], Simon Jackson[3], Anthony Williams-Jones[4]

[1] Department of Geology, University of Regina, Regina, Saskatchewan, Canada; [2] China University of Geosciences, Beijing, China; [3] Geological Survey of Canada, Ottawa, Ontario, Canada; [4] Department of Earth and Planetary Sciences, McGill University, Montreal, Canada

[ID]guoxiang.chi@uregina.ca; [ID]haixia.chu@cugb.edu.cn

Supplementary Information

Supplementary Table 1. Microthermometric results of fluid inclusions in quartz overgrowths from sandstones of the Athabasca Basin (those selected for LA-ICP-MS analysis are highlighted)

Sample #	Depth (m)	FI #	Occurrence	Size (μm)	V (%)	T_{fz} (ºC)	T_{m-HH} (ºC)	T_{m-ice} (ºC)	Salinity (wt. %)	$X_{(NaCl, wt)}$ (max)	T_h (ºC)
**WC-79-1 drill core**											
1404	63.4	c	Near D/O boundary	6	12	-92		-21.1	23.2	1.00	103
		e	Short trail in QO	8	10	-76h*		-21.3	23.4	0.98	123
		f		10	10	-71h*		-21.5	23.4	0.95	112
1406	112.5	a	Near D/O boundary	10	10			-27.5	25.5	0.41	167
		d	Near D/O boundary	6	10			-35.2	27.7	0.17	162
1407	138.0	b	Near D/O boundary	8	10			-25	24.7	0.58	155
		c	D/O boundary	10	10			-23.3	24.1	0.74	183
1408	170.6	b	D/O boundary	7	10	-58	-42.0	-25.2	23.2	0.10	125
1410	246.5	a	D/O boundary	6	12	-72	-35.5	-29.5	25.3	0.17	141
1416	364.3	a	D/O boundary	14	10			-11.1	15.1	1.00	129
		d	D/O boundary	20	12	-70h*	-29.8	-25.9	24.2	0.31	124
1419	440.1	c	D/O boundary	10	8	-86		-29.0	25.9	0.34	107
		d	D/O boundary	10	10	-88		-24.8	24.6	0.59	110
1421	475.5	b	D/O boundary	8	10		-33.9	-17.4	19.4	0.20	121
		d	D/O boundary	10	10	-71	-50	-25.6	23.3	0.06	120
		e	D/O boundary	10	12	-67		-20	22.4	1.00	164
		f	D/O boundary	10	10	-53		-19.2	21.8	1.00	62
1422	502.8	d	Isolated in QO	10	10			-21.1	23.2	1.00	103
1423	526.3	a	D/O boundary	24	10	-60		-20	22.4	1.00	78
		c	Near D/O boundary	18	10	-50		-17	20.1	1.00	61
1425	576.5	a	D/O boundary	8	10			-29.2	26.0	0.33	96
		b	D/O boundary	8	10	-90		-17	20.1	1.00	198
		c	D/O boundary	14	12	-72		-25.3	24.8	0.55	132
1426	593.1	a	Near D/O boundary	14	10	-72		-29.8	26.2	0.31	76
		b	D/O boundary	10	10	-95		-35.5	27.8	0.17	64
		c	D/O boundary	10	8	-84		-34.1	27.4	0.19	115
		d	Near D/O boundary	10	12	-74		-23.6	24.2	0.70	170
1427	601.2	a	D/O boundary	10	8	-89		-22.8	23.9	0.79	75
		c	D/O boundary	14	10	-55		-24.8	24.6	0.59	114
		e	D/O boundary	16	10				22.9***		69
		f	D/O boundary	12	10			-29.9	26.2	0.31	58
1428	613.7	a	D/O boundary	14	10				22.9***		66
		b	D/O boundary	10	10				22.9***		70
1429	647.2	a	D/O boundary	8	10	-71		-30.4	26.3	0.29	115
		b	D/O boundary	10	10	-67	T_s=218**	-22.5	32.6	0.90	50
1431	693.8	f	D/O boundary	12	10			-24.7	24.6	0.60	96
1432	747.4	a	D/O boundary	14	10				22.9***		64
		b	D/O boundary	18	10	-49		-23.3	24.1	0.74	65
		c	Near D/O boundary	12	10	-89		-31.5	26.7	0.25	84
		d	D/O boundary	10	10			-32.5	26.3	0.30	55

		e	D/O boundary	14	10	-74		-30.1	27.0	0.23	73
		a	D/O boundary	8	12	-80		-25.2	24.7	0.56	141
1433	768.9	b	D/O boundary	8	10		-51	-37.9	27.9	0.06	140
		c	Isolated in QO	10	12	-67		-31.5	26.7	0.25	149
1434	792.0	c	D/O boundary	12	10				22.9***		63
1435	805.0	b	D/O boundary	8	12			-34.2	27.4	0.19	162
		d	D/O boundary	6	12			-37.2	28.2	0.14	88
1436	845.8	d	Isolated in QO	8	10			-24.1	24.4	0.66	92
		a	D/O boundary	10	12			-24.9	24.6	0.59	135
		c	D/O boundary	12	10	-87		-33.1	27.1	0.21	57
1437	866.0	d	D/O boundary	8	10	-56		-24.8	24.6	0.59	52
		f	D/O boundary	10	10	-85h*		-44.3	29.9	0.08	110
		i	D/O boundary	16	10				22.9***		87
1438	892.5	c	D/O boundary	10	10			-25	24.7	0.58	85
		d	D/O boundary	12	10			-9.2	13.2	1.00	82
1438	892.5	f	D/O boundary	14	10			-25	24.7	0.58	72
**BL-08-01 drill core**											
1441	41.5	f	D/O boundary	10	12	-69		-28	25.6	0.39	153
		a	D/O boundary	10	10			-9.9	13.9	1.00	57
1442	57.2	b	D/O boundary	14	10		T_s=156**	-25.7	31.0	0.68	88
		e	D/O boundary	12	10	-70	T_s=179**	-28.2	32.9	0.61	89
1443	72.3	a	Isolated in QO	8	10	-71		-35.3	27.7	0.17	143
1444	88.0	c	D/O boundary	14	12	-75h*		-26.3	25.1	0.48	144
		a	D/O boundary	8	10			-19.6	22.1	1.00	65
		h	D/O boundary	7	10			-20.3	22.6	1.00	63
1446	132.0	i	D/O boundary	10	10			-31.9	26.8	0.24	67
		j	D/O boundary	12	10		T_s=189**	-24.6	31.9	0.76	50
		a	D/O boundary	12	12			-23	24.0	0.77	112
		b	D/O boundary	10	10			-25.6	24.9	0.53	114
1447	169.2	e	D/O boundary	12	10	-74	-42.2	-24.6	22.9	0.10	93
		f	D/O boundary	10	10				25.6***		78
		j	D/O boundary	10	10		T_s=141**	-30.3	32.1	0.52	132
		b	D/O boundary	16	12	-85		-37.2	28.2	0.14	127
1448	174.3	c	D/O boundary	14	12	-87		-35	27.7	0.17	129
		g	D/O boundary	8	10	-68	-42	-23.3	22.3	0.10	156
1449	199.3	f	Isolated in QO	8	10			-24	24.3	0.67	68
1450	214.5	e	Isolated in QO	10	10	-95		-32.8	27.0	0.22	93
		h	D/O boundary	8	10	-98		-30.1	26.3	0.30	89
1451	225.4	b	D/O boundary	8	10			-26.1	25.0	0.50	106
		a	D/O boundary	12	10				25.6***		107
		b	D/O boundary	12	10				25.6***		114
1453	267.7	e	D/O boundary	8	10				25.6***		58
		g	D/O boundary	6	10	-78		-42.5	29.5	0.09	148
		i	D/O boundary	8	10	-80	-41	-22.2	21.8	0.10	163
1455	291.0	a	D/O boundary	14	10	-93		-24.8	24.6	0.59	132

		e	D/O boundary	14	10	-87		-24.6	24.2	0.61	64
		f	D/O boundary	12	10				25.6***		77
1456	309.0	e	D/O boundary	12	12				25.6***		98
		f	D/O boundary	10	10	-87	-45	-24.3	22.7	0.08	172
1457	321.6	d	D/O boundary	8	10	-73	-35	-25.5	23.6	0.17	154
1459	347.1	h	D/O boundary	12	10				25.6***		108
		i	D/O boundary	12	10				25.6***		102
1470	670.1	a	D/O boundary	8	12	-66		-34.5	27.5	0.18	152
1473	717.2	a	D/O boundary	12	10	-80		-25	24.7	0.58	82
		h	D/O boundary	14	10				25.6***		
		i	D/O boundary	10	12	-63		-21.9	23.7	0.90	188
		j	D/O boundary	10	15	-53		-17	20.1	1.00	172
		l	D/O boundary	8	10	-69		-25.9	25.0	0.51	110
1474	748.5	a	D/O boundary	8	10	-70		-40.6	29.1	0.11	117
1477	828.4	i	D/O boundary	8	10	-61	-25.1	-18.2	20.4	0.57	154
1478	847.9	b	D/O boundary	8	10	-83		-28.5	25.8	0.36	84
		c	D/O boundary	8	10	95		-38.6	28.6	0.12	124
		d	D/O boundary	8	10	-72		-28.2	25.7	0.38	125
1479	866.6	c	D/O boundary	8	12	-90		-25.7	24.9	0.52	205
1479	866.6	d	D/O boundary	10	12	-87		-48.6	30.8	0.07	78
1480	883.0	a	D/O boundary	8	10	-74		-29.2	26.0	0.33	110
**DV10-001 drill core**											
1215	297.7	c	D/O boundary	8	10			-20.7	22.9	1.00	111
		d	Near D/O boundary	10	10		T_s=198**	-23.7	32.0	0.82	70
1216	313.8	a	Isolated in QO	6	10	-73		-21.5	23.4	0.95	154
		c	Cluster in QO	6	10	-79		-31	26.5	0.27	173
		d		6	10	-83		-26	25.0	0.50	182
1235	682.4	b	Isolated in QO	8	10	-58		-26	25.0	0.50	94
1237	714.1	c	D/O boundary	6	10	-62		-23.9	24.3	0.68	92
1237	714.1	d	D/O boundary	10	12	-77		-25.3	24.8	0.56	150
		e	D/O boundary	6	10	-65		-24	24.3	0.67	175
1238	726.65	a	D/O boundary	8	12	-98		-38	28.4	0.13	156
1239	741.8	a	Isolated in QO	8	10			-37.2	28.2	0.14	119
1241	778.3	c	D/O boundary	10	10	-75		-24.5	24.5	0.62	90
		d	D/O boundary	10	10	-70	-40.0	-22.5	22.0	0.11	79
		f	D/O boundary	10	10	-78	-42.3	-26.8	23.9	0.09	155
		g	Isolated in QO	7	10	-76		-24.9	24.6	0.59	72
1246	878.7	a	D/O boundary	12	10	-95		-38.1	28.5	0.13	117
		c	D/O boundary	10	10			-33.9	27.4	0.20	192
		d	D/O boundary	8	10	-77	-50	-25.5	24.8	0.54	198
1249	915.0	c	D/O boundary	8	10			-7.5	11.3	1.00	87
		e	D/O boundary	10	10			-28	25.6	0.39	98
1250	935.0	a	Cluster in QO	10	10			-25.2	24.7	0.56	85
		b		8	10			-27	25.3	0.44	76
1254	1021.3	b	D/O boundary	6	10	-75		-33.2	27.2	0.21	102

		d	D/O boundary	7	10	-70		-25.2	24.7	0.56	109
		f	D/O boundary	14	10	-91	-51	-33.8	26.5	0.06	173
1337	508.0	a	D/O boundary	7	10	-73		-38.2	28.5	0.13	100
		c	D/O boundary	12	10				26.0***		147
1340	561.2	a	D/O boundary	14	10			-31.8	26.8	0.25	79
		b	D/O boundary	16	10				26.0***		100
		e	D/O boundary	12	15	-79	-43.2	-34.5	26.9	0.09	203
1346	666.7	c	D/O boundary	6	10			-31.2	26.6	0.26	187
1349	756.3	a	D/O boundary	8	12	-69	-43.8	-22.5	21.9	0.08	178
**Rumpel Lake drill core**											
5302	27.9	c	Near D/O boundary	10	10			-20.3	22.6	1.00	62
5329	548.3	e	D/O boundary	6	10	-85	-42.0	-24.5	22.9	0.10	212
		g	D/O boundary	12	12				22.4***		210
		h	D/O boundary	10	10			-20.2	22.6	1.00	113
		j	D/O boundary	12	10	-70		-25	24.7	0.58	105
5330	570.7	i	D/O boundary	8	10	-82	-40	-25.2	23.2	0.11	134
		j	D/O boundary	8	10	-68		-26.7	25.2	0.46	142
		k	D/O boundary	12	15	-65		-2.3	3.9	1.00	129
5341	809.8	e	D/O boundary	10	10	-81		-24.5	24.5	0.62	69
5351	1029.9	d	D/O boundary	10	10	-86		-28.5	25.8	0.36	125
		g	D/O boundary	8	10	-90		-24	24.3	0.67	113
5361	1249.9	c	D/O boundary	8	10	-91	-40.0	-24.8	23.1	0.11	84
		d	Isolated in QO	10	10	-90h*	-50.0	-44.1	29.6	0.06	150
		h	D/O boundary	10	10	-90h*	-44.0	-35	27.1	0.08	168
		i	D/O boundary	10	10	-95	-40.0	-27.2	24.1	0.11	138
		j	D/O boundary	10	10	-84	-42.5	-32.7	26.3	0.09	130
5368	1403.6	d	D/O boundary	12	10				22.4***		123
		e	D/O boundary	16	10	-73		-9.7	13.7	1.00	78
		f	D/O boundary	12	10	-82		-18.2	21.1	1.00	68
		g	D/O boundary	16	10	-77	-38.1	-20.7	22.9	0.13	95
		i	D/O boundary	14	12				22.4***		98

T_{fz} – freezing temperature; $T_{m\text{-}HH}$ – hydrohalite-melting temperature; $T_{m\text{-}ice}$ – ice-melting temperature; Salinity – calculated from equation of Steele-MacInnis et al. (2011) using corresponding ice-melting temperature; $X_{(NaCl,\ wt)}$ (max) = wt(NaCl)/[wt(NaCl) + wt($CaCl_2$)], where wt is weight (the calculated X_{NaCl} ratios are maximum when $T_{m\text{-}HH}$ was not measured and $T_{m\text{-}ice}$ was used to estimate the ratios); T_h – homogenization temperature; h* – fluid inclusion was frozen during heating process; **halite-bearing fluid inclusions – salinities were calculated using the halite-melting temperatures (T_s) and corresponding ice-melting temperatures with the program by Steele-MacInnis et al. (2011); *** average salinity value from the drill core used for LA-ICP-MS quantification when salinity is unavailable for the specific inclusions; QO – Quartz overgrowth; D/O boundary – detrital-overgrowth boundary; Ref: Steele-MacInnis, M., Bodnar, R.J., Naden, J. 2011. Numerical model to determine the composition of H_2O-NaCl-$CaCl_2$ fluid inclusions based on microthermometric and microanalytical data. Geochim Cosmochim Acta 75: 21–40.

Supplementary Table 2. Concentrations (ppm) of elements in fluid inclusions analyzed by LA-ICP-MS

FI ID	Formation	Na23	Mg25	K39	Ca42	Fe57	U238	Na/(Na+Ca)
**WC-79-1 drill core**								
1426 a	Manitou Falls Formation	6,660	19,871	11,479	43,803	9,348	2.5	0.13
1425 c		32,820	12,418	17,401	25,696	5,927	1.6	0.56
1427 c		28,754	10,913	5,682		21,507	12.2	1.00
1431 f		10,357	29,951	13,412		13,200	16.3	1.00
1432 b		12,854	12,505	9,055		38,652	5.8	1.00
1432 c		38,802	17,241	4,557		5,732		1.00
1426 d		21,508	16,190	12,140		32,823		1.00
1427 e		4,271	14,449	6,139	47,826		2.1	0.08
1428 a		22,047	10,850	6,808	37,924	6,802	7.2	0.37
1428 b		3,277	19,171	5,974	40,990	8,442	26.8	0.07
1432 e		3,960	16,508	2,773	46,426	7,314	1.4	0.08
1432 d		5,822	17,309	19,153	35,087	6,597	2.8	0.14
1434 c		10,613	22,141	6,401	29,477	11,391	2.5	0.26
1437 c		13,169	17,930	3,688	35,585	2,924		0.27
1437 i		1,656	19,397	3,652	43,219	1,242		0.04
1438 d		15,941	13,590	5,553	39,370	28,515	4.7	0.29
1438 f		23,980	20,280	8,237	24,823	3,032	0.6	0.49
**BL-08-01 drill core**								
1441 f	Wolverine Point Formation	45,798	13,197	14,469			4.1	1.00
1442 b		6,718	15,497	54,525	28,424	3,713	12.7	0.19
1442 e			16,914	9,407	52,175	28,454	7.1	0.00
1444 c		45,320	9,633	56,326		17,088		1.00
1446 j		9,135	24,438	8,167				1.00
1447 a	Lazenby Lake Formation	4,295	19,742	7,920	40,905	3,000	5.0	0.10
1447 e		21,731	7,906	4,581				1.00
1447 f		3,592	23,509	6,379	42,610	48,282	9.1	0.08
1448 b		67,899	10,170	17,891		11,204	6.9	1.00
1448 c		8,447	27,659	5,389		33,347	8.9	1.00
1453 a		26,047	25,935	7,578	18,421	1,277	1.4	0.59
1453 b		15,292	22,696	4,004	34,969			0.30
1453 e			25,949	2,601	42,076			0.00
1455 e	Manitou Falls Formation	38,886	11,988	10,475		10,224	12.6	1.00
1455 f		39,551	15,356	19,456		5,306		1.00
1456 e		35,328	14,987	12,399		5,082		1.00
1456 f		14,606	13,328	11,963		13,175	3.7	1.00
1459 h		24,949	15,493	8,170	36,298	11,785	4.8	0.41
1459 i		30,747	10,500	14,198		35,978	7.1	1.00
1473 h		3,627	24,829	11,638	37,708	5,590	1.4	0.09
**DV10-001 drill core**								
1215 d	Lazenby Lake Formation	8,650	23,394	11,545	57,004	4,229	1.7	0.13
1337 c		48,454	11,117	16,941		43,490	17.3	1.00
1340 a		10,257	18,526	7,981	46,668	14,804	7.8	0.18
1340 b		3,624	19,697	9,504	48,641	7,558	2.2	0.07
1340 e		64,369	10,194	8,714		2,859	9.0	1.00
1241 c	Manitou Falls Formation	25,148	16,426	9,179	29,164	2,606	1.8	0.46
1241 d		24,887	4,214	7,997				1.00
1241 f			7,966	87,031		24,289	11.0	
1246 a		24,593	26,274	10,346		7,702		1.00
1254 f		37,980	16,271	7,212		27,534	5.3	1.00
**Rumple Lake drill core**								
5329 g	Manitou Falls Formation	46,302	9,739	19,095		11,536	10.8	1.00
5329 h		6,972	13,369	5,749	46,115	15,783		0.13
5329 i		32,733	14,170	13,161	18,163	23,970	5.7	0.64
5351d		34,168	4,814	31,504	33,058	44,477	18.9	0.51
5368 d		47,207	10,195	3,045	17,288	1,317		0.73
5368 e		3,258	12,662	3,140	21,372	2,304	3.6	0.13
5368 g		3,198	14,999	8,632	46,405	907	3.1	0.06
5368 i	Read Fm	17,258	15,318	7,286	32,769	5,616	2.0	0.34

Supplementary Table 3. Concentrations (ppm) of U in natural rocks and fluids

Rock or fluid types	Rock or fluid	U (ppm)	Data source(s)
Average sedimentary rocks	Arenite	1.5	Compiled by Kyser and Cuney (2015)
	Shales	4.2	
	Black-shales	53	
	Limestone	1.9	
Average igneous rocks	Tuffs	3	
	Granite/rhyolite	4.5	
	Syenite/phonolite	6.5	
	Andesite	0.79	
	Gabbro	0.84	
	Peridotite	0.01	
Average metamorphic rocks	Metamorphic rocks	3.5	
	Granulite	0.5	
Athabasca diagenetic fluids		0.6 – 26.8	This study
Athabasca U ore fluids		0.22 – 600	Richard et al. (2012, 2016)
Magmatic-hydrothermal fluid		0.31 – 97.6	Compiled by Richard et al. (2012)
Mid-ocean ridge fluids		3.8E-2 – 0.76	
Basement formation waters		1.9E-5 – 0.38	
Basin formation waters		3.1E-6 – 5.7E-2	
Sea water		3.3E-3	
River waters		6.9E-6 – 1.9E-3	
Geothermal fluids		1.9E-5 – 1.7E-3	

Date sources:

Kyser, K. & Cuney, M. 2015. Chapter 3: Geochemical characteristics of U and thorium and analytical methodologies. Mineralogical Association of Canada, Short Course Series 46, 39–84.

Richard, A., Rozsypal, C., Mercadier, J., Banks, D.A., Cuney, M., Boiron, M.-C. & Cathelineau, M. 2012. Giant U deposits formed from exceptionally U-rich acidic brines. Nature Geoscience 5, 142–146.

Richard, A., Cathelineau, M., Boiron, M.-C., Mercadier, J., Banks, D.A. & Cuney, M. 2016. Metal-rich fluid inclusions provide new insights into unconformity-related U deposits (Athabasca Basin and Basement, Canada). Mineralium Deposita 51, 249–270.
